# Case-Control Analysis of SNPs in GLUT4, RBP4 and STRA6: Association of SNPs in STRA6 with Type 2 Diabetes in a South Indian Population

**DOI:** 10.1371/journal.pone.0011444

**Published:** 2010-07-06

**Authors:** Anup Kumar Nair, Divya Sugunan, Harish Kumar, Gopalakrishnapillai Anilkumar

**Affiliations:** 1 Amrita School of Biotechnology, Amrita Vishwa Vidyapeetham, Kollam, Kerala, India; 2 Amrita Institute of Medical Science, Amrita Vishwa Vidyapeetham, AIMS Kochi, Kerala, India; Innsbruck Medical University, Austria

## Abstract

**Background:**

The inverse relationship between GLUT4 and RBP4 expression is known to play a role in the pathogenesis of type 2 diabetes. Elevated levels of RBP4 were shown to cause insulin resistance in muscles and liver. Identification of STRA6 as a cell surface receptor for RBP4 provides further link in this axis and hence we analyzed SNPs in these three genes for association with type 2 diabetes in a South Indian population.

**Methodology/Principal Findings:**

Selected SNPs in the three genes were analyzed in a total of 2002 individuals belonging to Dravidian ethnicity, South India, by Tetra Primer ARMS PCR or RFLP PCR. Allele frequencies and genotype distribution were calculated in cases and controls and were analyzed for association by Chi-squared test and Logistic regression. Haplotype analysis was carried out for each gene by including all the markers in a single block. We observed a significant association of three SNPs, rs974456, rs736118, and rs4886578 in *STRA6* with type 2 diabetes (P = 0.001, OR 0.79[0.69–0.91], P = 0.003, OR 0.81[0.71–0.93], and P = 0.001, OR 0.74[0.62–0.89] respectively). None of the SNPs in *RBP4* and *GLUT4* showed any association with type 2 diabetes. Haplotype analysis revealed that two common haplotypes H1 (111, P = 0.001, OR 1.23[1.08–1.40]) and H2 (222, P = 0.002 OR 0.73[0.59–0.89]) in *STRA6*, H6 (2121, P = 0.006, OR 1.69[1.51–2.48]) in *RBP4* and H4 (2121, P = 0.01 OR 1.41[1.07–1.85]) in *GLUT4* were associated with type 2 diabetes.

**Conclusion:**

SNPs in *STRA6*, gene coding the cell surface receptor for RBP4, were significantly associated with type 2 diabetes and further genetic and functional studies are required to understand and ascertain its role in the manifestation of type 2 diabetes.

## Introduction

Type 2 diabetes is primarily characterized by insulin resistance, relative insulin deficiency and fasting hyperglycemia [1]. Adipocytes, muscle tissue and liver are the main insulin responsive sites in the body. Adipocytes and muscles contain an insulin sensitive glucose transporter, GLUT4, and are the major glucose disposal sites in response to insulin, whereas insulin inhibits the process of endogenous glucose production by liver. This concerted action of insulin on adipocytes, muscles and liver helps in maintaining blood glucose homeostasis [1], [2]. It has been reported that there is an adipocyte specific downregulation of GLUT4 in type 2 diabetes. This decrease in GLUT4 expression is concomitant with an increased expression of retinol binding protein 4 (RBP4) [3], [4]. RBP4, expressed in adipocytes and liver, is an adipokine involved in the transport of vitamin A/retinol. Apart from its role as a retinol transporter, RBP4 has also been implicated in causing insulin resistance in muscle and liver [3]. Additionally several studies in various populations have reported elevated levels of RBP4 in type 2 diabetes and related metabolic parameters [5]–[7]. It was shown that RBP4 modulates the activity of phosphoenolpyruvatecarboxykinase (PEPCK) in liver subsequently altering gluconeogenesis [3]. In muscles, RBP4 alters the activation of Phophoinositide-3-kinase (PI3K) in response to insulin thus decreasing insulin stimulated glucose uptake into muscles [3]. The recent identification of Stimulated by Retinoic Acid gene homolog 6 (STRA6) as the high affinity cell surface receptor for RBP4 may give us further insight into the mode of action of RBP4 [8]. STRA6 is a multi transmembrane domain protein that mediates the cellular uptake of vitamin A. In accordance with its role as the transmembrane transporter of vitamin A, STRA6 plays a major role in embryonic development [8], [9]. Studies have shown that STRA6 is overexpressed in colon cancer [10]. But neither functional nor genetic studies have been carried out to identify how STRA6 may play a role in imparting the effect of RBP4 on muscle and liver insulin resistance and subsequently type 2 diabetes.

Genetic studies have been carried out with respect to single nucleotide polymorphisms (SNPs) in *GLUT4* and *RBP4* but with varied results [11]–[19]. Though association studies pertaining to SNPs in RBP4 with type 2 diabetes and related parameters have been done in many populations, detailed studies of SNPs in *GLUT4* with type 2 diabetes is lacking. SNPs in both *RBP4* and *GLUT4* have not been analyzed in Indian population for their association with type 2 diabetes. With respect to *GLUT4*, a study in Welsh population identified a silent polymorphism (Asn130) in exon-4 present in diabetic subjects. They also identified a Val383Ile mutation present in 3 out of 160 type 2 diabetes subjects but in none of the control subjects. But further analysis in British population failed to confirm any association of these mutations with type 2 diabetes [15]–[16]. Other studies in *GLUT4* include RFLP studies with KpnI enzyme in Italian, Caucasians, Chinese, and Asian Indians, American black and Japanese population [17]–[18]. These studies failed to establish an association between the KpnI allele and type 2 diabetes. None of these studies focused on SNPs present in the promoter and coding region of *GLUT4.* Recent studies in other populations have identified common variants and haplotypes in *RBP4* to be associated with type 2 diabetes [11], [13]. Other studies have also identified SNPs in *RBP4* to be associated with altered serum lipid levels (12,14). Interestingly, none of these studies looked at polymorphisms in *STRA6*; the only identified high affinity receptor for RBP4.

The triad of *GLUT4*-*RBP4-STRA6* may play an important role in the pathogenesis of type 2 diabetes, wherein the adipocyte specific down regulation of GLUT4 leads to an increased expression of RBP4 and this increase in RBP4 expression leads to insulin resistance via STRA6. In this study, SNPs in *GLUT4, RBP4 and STRA6* were analyzed for association with type 2 diabetes in a South Indian population. We report for the first time that genetic variants in *STRA6* are significantly associated with type 2 diabetes.

## Results

### Characteristics of the study population

The study was conducted by using DNA isolated from blood samples of 2002 unrelated individuals (1002 cases with type 2 diabetes and 1000 normoglycemic control subjects) belonging to Dravidian ethnicity from Kerala, South India. Diabetes related clinical and biochemical parameters were collected for all the case samples whereas fasting blood glucose, BMI and blood pressure were documented for control samples. Clinical and biochemical features of the study population are summarized in Table 1.

**Table 1:** Clinical Characterization of the study population.

	Subjects with type 2 diabetes	Subjects without type 2 diabetes
Number of subjects	1002	1000
Sex (Male/Female)	584/418	470/530
Age (Years)	55.7±10.2	51.07±10.2
Age of onset of diabetes (years)	45.08±9.04	-
BMI (Kg/m^2^)	25.05±3.4	23.09±3.9
Fasting Blood Glucose (mmol/L)	8.67±3.4	4.9±0.58
HBA1C (%)	8.3±1.8	-

Data represented as mean±SD.

### Test for Hardy-Weinberg equilibrium and LD analysis

Test for Hardy-Weinberg equilibrium suggested that the genotypes for all the SNPs except rs351224 (*STRA6*) were in Hardy-Weinberg proportions and there was no deviation from Hardy-Weinberg equilibrium. rs351224 was subsequently omitted from further analysis. dbSNP ID, Minor allele frequency (MAF) and HWE P value for all the selected SNPs are summarized in Table 2. LD analysis using Haploview revealed that the 2 SNPs in *STRA6, rs736118* and rs4886578 spanned one LD block (D' = 0.95), rs974456 was not in LD with the two SNPs and rs351224 was omitted from the analysis due to deviation from HWE. Kovacs et al have previously defined the LD pattern for *RBP4* for Caucasian population [12]. In our population also we found a similar LD pattern with SNPs rs3758539, rs36014035 and rs34571439 spanning one LD block. rs3758538 was not in LD with any of the above SNPs. In case of *GLUT4,* the 4 SNPs spanned two different LD blocks with SNPs rs2654185 and rs5412 in one LD block and SNPs rs5418 and rs5435 in the second LD block.

**Table 2:** Summary of selected SNPs.

dbSNP ID	Gene	Position	MAF	HWE P value (Controls)	HWE P value (Cases)
rs974456	*STRA6*	Intron 7	0.27	0.18	0.54
rs351224	*STRA6*	Intron 7	0.43	3.5×10^−5^	0.002
rs736118	*STRA6*	Exon 17	0.32	0.14	0.45
rs4886578	*STRA6*	Intron 18	0.14	0.074	0.9
rs3758538	*RBP4*	promoter	0.18	0.37	0.8
rs3758539	*RBP4*	promoter	0.33	0.43	0.64
rs36014035	*RBP4*	Intron 4	0.42	0.96	0.49
rs34571439	*RBP4*	3′UTR	0.35	0.23	0.07
rs2654185	*GLUT4*	promoter	0.37	0.14	0.07
rs5412	*GLUT4*	promoter	0.30	0.12	0.52
rs5418	*GLUT4*	5′UTR	0.38	0.42	0.29
rs5435	*GLUT4*	Exon 4	0.30	0.59	0.62

MAF – minor allele frequency in the whole study group. HWE – Hardy Weinberg Equillibrium. SNPs were selected from dbSNP, NCBI based on their position in the gene, minor allele frequency or previous studies. Hardy Weinberg test was done using the Pearson's goodness of fit test and a P value <0.05 was considered to show significant deviation of the observed genotypes from Hardy-Weinberg proportions.

### Association analysis of genetic variants in *GLUT4*, *RBP4* and *STRA6* with type 2 diabetes

All the three SNPs in *STRA6* viz. rs974456, rs736118 and rs4886578 were significantly associated with type 2 diabetes (*P* = 0.001, OR 0.79[0.69–0.91], *P* = 0.003, OR 0.81[0.71–0.93], and *P* = 0.001 OR 0.74[0.62–0.89] respectively). Significant *P* values were also obtained in the additive model analysis after adjustment for age, sex and BMI. The associations remain statistically significant even after correction for multiple testing by Bonferroni correction (×12, *P* value 0.012, 0.036, and 0.012 respectively). As revealed by ODD's Ratio (OR), all three SNPs in *STRA6* tend to be associated with a decreased susceptibility for type 2 diabetes. None of the selected SNPs in *GLUT4* or *RBP4* showed any significant association with type 2 diabetes. A silent polymorphism in *GLUT4*, rs5435, showed a slight trend towards association (P = 0.05). Results of association analysis are summarized in Table 3.

**Table 3:** Association analysis of selected SNPs in *STRA6*, *RBP4* and *GLUT4* with type 2 diabetes.

SNP ID	Gene	Allele major/minor	MAF		OR (95%CI)	P/P*	*P*(add)
			Cases	Control			
rs974456	*STRA6*	*C/T*	0.25	0.30	0.79(0.69–0.91)	**0.001/0.012**	**0.003**
rs736118	*STRA6*	*G/A*	0.30	0.35	0.81(0.71–0.93)	**0.003/0.036**	**0.01**
rs4886578	*STRA6*	*G/A*	0.12	0.16	0.74(0.62–0.89)	**0.001/0.012**	**0.0009**
rs3758538	*RBP4*	*A/C*	0.19	0.18	1.06(0.9–1.2)	0.43/NS	0.63
rs3758539	*RBP4*	*G/A*	0.33	0.35	0.91(0.79–1.04)	0.18/NS	0.48
rs36014035	*RBP4*	*T/G*	0.43	0.42	1.02(0.9–1.16)	0.71/NS	0.18
rs34571439	*RBP4*	*T/G*	0.35	0.36	0.93(0.82–1.07)	0.35/NS	0.63
rs2654185	*GLUT4*	*C/A*	0.37	0.36	1.01(0.89–1.15)	0.83/NS	0.5
rs5412	*GLUT4*	*G/A*	0.29	0.31	0.88(0.76–1.01)	0.07/NS	0.05
rs5418	*GLUT4*	*A/G*	0.37	0.40	0.89(0.77–1.02)	0.09/NS	0.25
rs5435	*GLUT4*	*G/A*	0.29	0.32	0.87(0.76–1.00)	0.05/NS	0.1

MAF- minor allele frequency, OR- ODD's ratio. *P*- P value calculated by Chi Squared test for difference in allele frequency between cases and controls. * - P value after Bonferroni correction, *P*(add) – P value obtained by logistic regression after adjustment for age, sex and BMI using an additive model. *P* values showing significant association have been indicated in bold. NS- Not significant.

### Haplotype analysis of SNPs in *GLUT4*, *RBP4* and *STRA6*

In case of *STRA6*, rs351224 was not included in the haplotype analysis because of significant deviation from Hardy-Weinberg proportions (Table 2). Therefore, only three SNPs in the order rs974456, rs736118 and rs4886578 were used as a single block. Five common haplotypes in *STRA6* with a frequency >5% accounted for 97% of the observed haplotypes in case subjects. Analysis of these haplotypes in cases and controls revealed that the haplotype H1 (GGC, 111) and H2 (AAT, 222) were significantly associated with type 2 diabetes (*P* = 0.001 and *P* = 0.002 respectively) even after correction for multiple testing (*P* = 0.005 and P = 0.01) (Table 4). The haplotype H1 (GGC) had a higher frequency in patients (61.8%) than in controls (56.9%), whereas the haplotype H2 (AAT) was observed more in controls (12.5%) than in cases (9.4%) indicating that the minor alleles (A,A and T) of rs736118, rs4886578 and rs974456 are protective against type 2 diabetes. Haplotype analysis of SNPs in *RBP4* revealed that six common haplotypes with a frequency >3.5% in type 2 diabetic patients accounted for 90% of the observed haplotypes. A case-control analysis of these haplotypes showed that the haplotype H6 (2121, CGGT) in the order rs3758538, rs3758539, rs36014035 and rs34571439 is significantly associated (*P* = 0.006) with type 2 diabetes (Table 5). This association remains significant even after correction for multiple testing (*P* = 0.036). When we omitted rs3758538 from the haplotype analysis due to low minor allele frequency, we found that the haplotype (121) was significantly associated with type 2 diabetes with a *P* = 0.002 (data not shown). We also analyzed the haplotypes in *GLUT4* for association with type 2 diabetes. 87.7% of the total haplotypes were constituted by four common haplotypes with a frequency >5% in case samples. Case-control analysis revealed that the haplotype H4 (2121, AGGG) defined by SNPs rs2654185, rs5412, rs5418 and rs5435 is marginally associated with type 2 diabetes after correction for multiple testing (P/P* = 0.01/0.04). Results of haplotype analysis with SNPs in *GLUT4* are summarized in Table 6.

**Table 4:** Haplotype analysis of selected SNPs in *STRA6.*

	Haplotypes	Type 2 diabetes patients (%)	Non diabetic controls (%)	OR (95%CI)	P/P* values
H1	111	61.8	56.9	1.23(1.08–1.40)	**0.001/0.005**
H2	222	9.46	12.5	0.73(0.59–0.89)	**0.002/0.01**
H3	121	9.67	9.43	1.03(0.83–1.27)	0.76/NS
H4	221	9.06	9.78	0.92(0.74–1.14)	0.45/NS
H5	211	7.06	7.89	0.88(0.70–1.12)	0.33/NS

Haplotype frequencies were compared using Chi squared test. Haplotypes are defined by selected SNPs in *STRA6* in the following order – rs974456, rs736118 and rs4886578. rs351224 was not included in the haplotype analysis. * P values after Bonferroni correction. NS- not significant, 1-major allele, 2- minor allele.

**Table 5:** Haplotype analysis of selected SNPs in *RBP4.*

	Haplotypes	Type 2 diabetes patients (%)	Non diabetic controls (%)	OR (95%CI)	P/P* values
H1	1111	48.6	48.3	1.01 (0.89–1.16)	0.77/NS
H2	1222	20.6	22.2	0.91 (0.78–1.07)	0.27/NS
H3	2222	7.3	7.4	1.0 (0.78–1.28)	0.99/NS
H4	2111	5.0	5.5	0.9 (0.68–1.21)	0.51/NS
H5	1121	4.6	3.5	1.31 (0.94–1.81)	0.1/NS
H6	2121	3.8	2.2	1.69 (1.51–2.48)	**0.006/0.036**

Haplotype frequencies were compared using Chi squared test. Haplotypes are defined by selected SNPs in *RBP4* in the following order – rs3758538, rs3758539, rs36014035 and rs34571439. * P values after Bonferroni correction. NS- not significant, 1-major allele, 2- minor allele.

**Table 6:** Haplotype analysis of selected SNPs in *GLUT4.*

	Haplotypes	Type 2 diabetes patients	Non diabetic controls	Chi squared	P/P* values
H1	1111	31.5	29.0	1.12 (0.96–1.30)	0.12/NS
H2	1211	24.8	26.0	0.93 (0.80–1.09)	0.41/NS
H3	2122	23.4	26.0	0.86 (0.74–1.01)	0.07/NS
H4	2121	8.0	5.8	1.41 (1.07–1.85)	**0.01/0.04**

Haplotype frequencies were compared using Chi squared test. Haplotypes are defined by selected SNPs in *GLUT4* in the following order – rs2654185, rs5412, rs5418 and rs5435. *- *P* values after Bonferroni correction. NS- not significant, 1-major allele, 2- minor allele.

**Figure 1:**
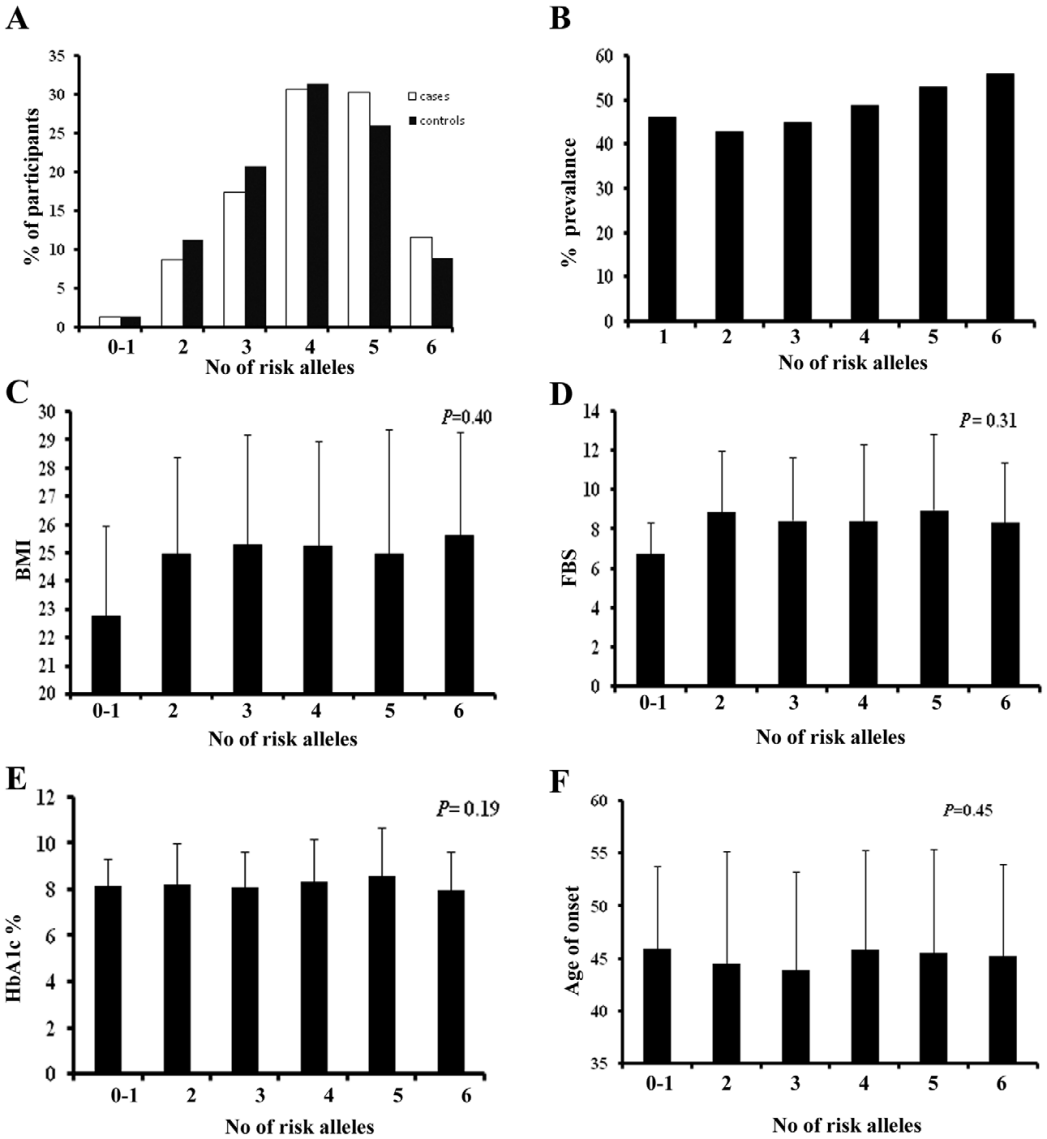
Combined effect of increasing number of risk alleles from STRA6 (rs736118), RBP4 (rs3758539) and GLUT4 (rs5435). A. risk allele distribution in subjects with and without type 2 diabetes. B. percentage prevalence of type 2 diabetes in the study population with increasing number of risk alleles. C, D, E and F. Association analysis of BMI, fasting blood glucose, HBA1C and age at onset of diabetes with increasing number of risk alleles.

Recent studies in other populations have reported a significant association of SNPs in *RBP4* with serum lipid levels [12]–[14]. We also analyzed whether the promoter SNPs in RBP4 (rs3758538 and rs3758539) are associated with diabetes related phenotype in our population. We did not observe a significant association of these SNPs with serum lipid levels or with other analyzed traits (data not shown). Analysis of SNPs in *STRA6* also did not reveal any significant association with type 2 diabetes related phenotypes (data not shown). We also did not find any evidence of multiplicative gene-gene interaction in modulating BMI, fasting blood glucose, HBA1C and age of onset of diabetes though a higher proportion of individuals with diabetes had higher number of risk alleles (Fig. 1). Percentage prevalence of diabetes increased with increase in number of risk alleles when analyzed with sentinel SNPs in *STRA6* (rs736118), *RBP4* (rs3758539) and *GLUT4* (rs5435).

## Discussion

In this study, we used a case-control approach for analyzing SNPs in three candidate genes viz. *GLUT4*, *RBP4* and *STRA6* for association with type 2 diabetes. We provide evidence that SNPs in STRA6 are significantly associated with type 2 diabetes. We also identified two common haplotypes in *STRA6* associated with type 2 diabetes. Furthermore, common haplotypes in *GLUT4* and *RBP4* were also found to be associated with type 2 diabetes. After a case-control analysis in 1002 type 2 diabetes samples and 1000 control samples, we found that the three SNPs in STRA6, rs736118 (P = 0.003), rs4886578 (P = 0.001) and rs974456 (P = 0.001) were significantly associated with type 2 diabetes. rs736118 is a G>A polymorphism leading to a Met to Ile change in the C-terminal end of STRA6. Though Met and Ile are hydrophobic amino acids, evidence from literature suggests that such changes can alter the trafficking and cell surface expression of proteins. For example, a V302M mutation in the cytoplasmic G loop of Kir6.2 (*KCNJ2*) led to a loss of coassembly with the wild type protein thereby affecting protein trafficking to the cell surface [20]. Alternatively, it was also shown that this mutation can modulate the potassium ion conductance by Kir6.2 [21].

rs4886578 (G>A), is an exon-intron boundary polymorphism whereas rs974456 (C>T) is present in intron 7. It has been shown that SNPs present in the non-coding region can modulate gene expression and SNPs present in exon-intron boundary can alter splice forms [22]. Thus, both these variations can lead to differential expression of the protein. Subsequent haplotype analysis revealed two haplotypes (H1-CGG and H2- TAA, in the order rs974456, rs736118 and rs4886578) to be significantly associated with type 2 diabetes (P = 0.001 and P = 0.002). Haplotype H1 was present more in type 2 diabetic patients (61.8%) than normoglycemic controls (56.8%) whereas the haplotype H2 was present more in normoglycemic controls (12%) than in cases (9.4%). This indicates that the haplotype H2 which is composed of the minor alleles in the 3 SNPs decreases the susceptibility to type 2 diabetes. These findings support the hypothesis that STRA6 acts as a putative link between RBP4 and its effect on muscle, liver and whole body insulin resistance. Additionally it was reported that Wnt-1 and retinoic acid synergistically induce the expression of STRA6 [10]. It is interesting to note that TCF4, a transcription factor, coded by *TCF7L2*, the most strongly associated and replicated type 2 diabetes susceptibility loci, is an integral part of Wnt-1 signaling [23]–[29]. TCF4 is known to interact with β-catenin and induces several wnt-1 signalling target genes, and it is possible that TCF4 also induces the expression of STRA6. The role of STRA6 in type 2 diabetes and its regulation by TCF4 are currently under investigation in our laboratory.

We also analyzed SNPs in *RBP4* and *GLUT4* for association with type 2 diabetes and related parameters. SNPs in *RBP4* have been previously reported to be associated with type 2 diabetes in Mongolian population [11]. They identified a functional variant (rs3758539) in the promoter of *RBP4* associated with type 2 diabetes. Another study which analyzed SNPs in the *RBP4* for association with type 2 diabetes identified a common haplotype associated with type 2 diabetes but failed to observe any single SNP association [12]. We also did not observe any significant association of SNPs in *RBP4* but our haplotype analysis revealed that a common haplotype (H6 – CGGT in the order rs3758538, rs3758539, rs36014035 and rs34571439) is significantly associated with type 2 diabetes (P = 0.004). Kovacs et al. identified significant association of the common haplotype AGGTGC (defined by the order rs3758538, rs3758539, rs10882283, c.248+44T>C, rs1226584 and rs10882273) with type 2 diabetes [12]. They also reported that the SNP rs1226584 is in complete LD with rs34571439, and rs10882273 is in complete LD with rs34571439. Thus, the haplotype association we identified is comparable to the one reported in the above study except for rs3758538. This may be due to the lower minor allele frequency of rs3758538 and comparably similar frequencies in cases and controls. Other genetic studies with SNPs in *RBP4* have reported association with serum lipid parameters but in our study we failed to observe any significant association of the promoter polymorphisms with serum lipid levels or with other diabetes related parameters [13], [14]. RBP4 has also been implicated in diabetic retinopathy with enhanced expression in cases with proliferative diabetic retinopathy when compared to non–proliferative diabetic retinopathy or no retinopathy [30]. However, in this study, we did not observe any significant association of SNPs in *RBP4* and *STRA6* with diabetic retinopathy in a case control analysis using 155 samples with diabetic retinopathy and 150 samples with type 2 diabetes for more than 12 years with no symptoms of diabetic retinopathy.

Although *GLUT4* is a strong candidate gene for diabetes related studies, we did not find any evidence of individual SNPs in *GLUT4* associated with type 2 diabetes in our population. Haplotype analysis with selected SNPs revealed a common haplotype 2121 defined by the order rs2654185, rs5412, rs5418 and rs5435 marginally associated with type 2 diabetes. We also found that the SNP rs5435 showed an increasing trend towards association with considerable difference in minor allele frequency in cases and controls. Probably this SNP in *GLUT4* may increase the susceptibility to type 2 diabetes in combination with other SNPs. It should be noted that the sample size used in this analysis is suitable for stronger effects as found in *STRA6* but smaller effects like those observed in GLUT4 (rs5435) may be missed and analysis of a larger sample size may confirm such effects. Our gene-gene interaction data reveals that when the sentinel SNPs in *STRA6*, *RBP4* and *GLUT4* are combined together they increase the prevalence of type 2 diabetes with increase in the number of risk alleles (Figure 1B). This indicates that the three genes may play a combined role in increasing the susceptibility of type 2 diabetes. But this increase in number of risk alleles had no effect on BMI (Figure 1C), fasting blood glucose (Figure 1D) or HBA1c (Figure 1E) indicating that the increase in the susceptibility of type 2 diabetes is modulated through some other pathway most probably by affecting the measure of insulin resistance.

Recent Genome Wide Association Studies (GWAS) in different populations have identified various type 2 susceptibility genes [31]–[34]. But most of these studies have been done in Western population and only few GWAS studies have been carried out in Asian population, notably in Japanese and Han Chinese population [35]–[36]. Most of the type 2 susceptibility genes identified by GWAS in Western population play a role in insulin secretion which correlates well with the type 2 diabetes phenotype in that population. GWAS study in Japanese population also led to the identification of type 2 diabetes susceptibility genes similar to that found in Western population but with greater effect size and explained variance [35]. GWAS study in Japanese population also identified *KCNQ1* as a diabetes susceptibility loci which was further confirmed in other population [37]–[38]. Similar GWAS studies in Han Chinese led to the confirmation of the common type 2 succeptibility genes observed in Western and Japanese population with the addition of two more novel type 2 diabetes susceptibility loci. The study identified *PTPRD* and Serine Racemase (*SRR*) as novel type 2 diabetes susceptibility genes not observed in Japanese and Western population [36].

GWAS data for type 2 diabetes in South Indian population is still not available. India has the highest number of cases with type 2 diabetes and is referred as the diabetic capital of the world with 58.4 million people with type 2 diabetes. Indian population differs from other population in certain unique biochemical and clinical parameters like increased insulin resistance, higher adiposity, lower adiponectin levels and higher levels of sensitive C-reactive protein [39]. This indicates that in Indian population the adipose tissue mass in the form of central obesity plays a crucial role in the pathogenesis of type 2 diabetes. Similarly genetic factors also differ in their effect on type 2 diabetes as compared to European population. None of the GWAS study in other populations identified STRA6 as a susceptibility loci for type 2 diabetes. Since no data was available for South Indian population in HapMap we compared LD pattern of the recently included Gujarati Indian population from Houston, Texas (GIH) with Japanese (JPT), Han Chinese (CHB) and western population (CEU) for differences in LD pattern of the STRA6 gene. We found that the LD pattern in GIH population was different from other populations.

In conclusion, we analyzed SNPs in three candidate genes namely, *GLUT4*, *RBP4* and *STRA6* for association with type 2 diabetes. We provide evidence supporting a novel genetic role for the *STRA6* loci in type 2 diabetes. A significant association was observed between two common haplotypes in *STRA6* and type 2 diabetes. Additionally haplotype analysis with SNPs in *RBP4* and *GLUT4* also revealed significant association of a common haplotype in both genes with type 2 diabetes.

## Materials and Methods

### Ethics statement

Written informed consent forms were obtained from all the participating subjects. The study was conducted following the guidelines of Indian Council of Medical Research and approved by the ethics committee at AIMS.

### Case and Control samples

The study was conducted using samples from 2002 unrelated individuals from Kerala, South India as a part of an ongoing project, Amrita Diabetes Awareness and Welfare Study. Case samples were collected from Endocrinology department, Amrita Institute of Medical Sciences (AIMS), Kochi, Kerala. All samples were collected following the guidelines of American Diabetes Association with fasting blood glucose >120 mg/dl and/or 2-hour plasma glucose >200 mg/dl. Case samples were collected after detailed investigation of medical records for symptoms, use of oral hypoglycemic agent and/or insulin, measurement of fasting blood glucose and other related anthropometric parameters. Subjects diagnosed with type 2 diabetes after the age of 60 or subjects who were started on insulin therapy within one year of diagnosis were excluded from the study. Subjects with type 1 diabetes, family history of type 1 diabetes, maturity onset diabetes of the young, monogenic forms of diabetes, or drug induced diabetes were also excluded from the study. Samples from patients outside Kerala and patients migrating from other parts of the country were not included. Case reports of diabetic nephropathy, diabetic retinopathy and fatty liver disease as diagnosed by the physician were also documented during collection.

Age, sex and ethnicity matched normoglycemic control subjects were recruited in the study by public advertisement and increased awareness by offering screening for diabetic risk factors at specially organized medical camps. All the participants of these medical camps were subjected to a health questionnaire for detailed investigation of their disease status, family history, socio-economic status, food habits, smoking and alcohol status, BMI and blood pressure (BP). The inclusion criteria for the healthy controls were: a) above 40 years of age, b) no family history of diabetes, c) not taking any oral hypoglycemic agent or insulin, d) blood glucose levels <110 mg/dl. Clinical characterization of the study subjects is summarized in the Table 1.

### Genotyping

Genomic DNA was isolated from peripheral blood leukocytes by salting out procedure [40]. SNP genotyping was performed by Tetraprimer Amplification Refractory Mutation System PCR (Tetraprimer ARMS PCR) and Restriction Fragment Length Polymorphism PCR (RFLP PCR) [41]. A total of 12 SNPs from *STRA6*, *RBP4* and *GLUT4* were selected from dbSNP, NCBI, either based on their position in the gene viz. 5′ UTR, exon, exon-intron boundary, 3′UTR and minor allele frequency (MAF, >0.05) reported in dbSNP or based on previous association studies. All the novel SNPs were selected based on their position to influence the expression or function of the protein. rs5435 in *GLUT4* has been previously studied in European population. In addition, we selected three more SNPs in *GLUT4* based on position and minor allele frequency as reported in dbSNP, NCBI. For *RBP4*, we selected SNPs based on previous studies in European population whereas for *STRA6*, SNPs were selected based on their reported population diversity data and position in the gene. There is only one reported non- synonymous SNP, rs736118, in *STRA6* with population diversity data and a MAF >0.05 in Asian population so only this SNP from the coding region of *STRA6* was included in the study. Other SNPs selected from *STRA6* include rs4886578 present in the intron-exon boundary, rs974456 and rs351224 present in the intron. All SNPs were genotyped by tetraprimer ARMS PCR and RFLP PCR. Genotyping success rate was generally >96% except for rs5418 in *GLUT4* where the success rate was >87%. A summary of selected SNPs is given in Table 2. All SNPs except rs736118 (*STRA6*), rs974456 (*STRA6*) and rs5418 (*GLUT4*) were genotyped by Tetraprimer ARMS PCR. Genotyping plates which showed amplification in negative control (without template DNA) were repeated. All ambiguous genotypes were also repeated in duplicates till clear genotypes were available. Approximately 10% (n = 194) samples were re-genotyped for cross validation and the genotype success rate was >99.5%. rs736118, rs974456 and rs5418 were genotyped by RFLP-PCR using the restriction enzymes Nco1, Xho1 and BamH1 respectively. In case of rs736118 (Nco1) and rs974456 (Xho1) the presence of minor allele resulted in the lack of restriction enzyme site and hence there was no change in the PCR amplicon after restriction digestion. So to ensure the genotyping accuracy all samples homozygous for the minor allele were selectively re-genotyped. Randomly selected 10% samples were genotyped for these SNPs also and the genotyping accuracy was found to be >99.5%. All DNA amplicons and fragments were analyzed by agarose gel electrophoresis and visualized by ethidium bromide staining in gel documentation system, Biorad, USA. PCR primer designs and reaction conditions are available on request.

### Statistical analysis

Hardy Weinberg equilibrium (HWE) test was done for each SNP by Pearson's Goodness of fit Chi square test before further analysis. Association analysis of each SNP with type 2 diabetes was done by Chi-square test, for difference in allele frequency between cases and controls. Association of SNPs with type 2 diabetes was also confirmed by logistic regression analysis after adjustment for age, sex and BMI. An additive model was used for logistic regression where homozygotes for the major allele (1_1), heterozygotes (1_2) and homozygotes for the minor allele (2_2) were coded to a continuous numeric variable for the genotype as 0, 1 and 2 respectively. Linkage disequilibrium was estimated for combined data of cases and controls using Haploview version 4.1. Haplotype analysis was done by using the software SNP and Variation Suite Ver-7 from Golden Helix, Inc. Haplotype analysis was done by using all the markers in a gene as a single block. Quantitative traits were analyzed for association with type 2 diabetes by ANCOVA (available at http://faculty.vassar.edu/lowry/VassarStats.html) and multivariate regression analysis after adjustment for age, sex and BMI. Correction for multiple testing was done by Bonferroni's inequality method wherever applicable and was defined as *P* value (single tests) × number of tests. Bonferroni correction was not done for multiple traits due to lack of any significant association. Multiallelic analysis with sentinel SNPs in STRA6 (rs736118), RBP4 (rs3758539) and GLUT4 (rs5435) was done by ANOVA after categorizing the participants into groups according to the number of risk alleles they carried. All association analysis was performed using the software SNP and Variation Suite Ver-7 from Golden Helix, Inc.
